# Supplementary material for: Investigating the Pathogenic Interplay of Alpha-Synuclein, Tau, and Amyloid Beta in Lewy Body Dementia: Insights from Viral-Mediated Overexpression in Transgenic Mouse Models
Source: Biomedicines. 2023 Oct 22;11(10):2863. doi: 10.3390/biomedicines11102863 (PMC10604054; doi:10.3390/biomedicines11102863)
Supplement: Supplementary file 1 [file biomedicines-11-02863-s001.zip › biomedicines-2661942-supplementary.pdf]

biomedicines

MDPI

Supplementary Materials

Supplementary Table S1. Behavioral statistics of 6- and 9-MPI hTau overexpressing human αsyn. Statistics for each behavioral test and their corresponding measured parameters (open field analysis (OFA), elevated plus maze (EPM), contextual/cued fear conditioning (CFC)). All behavioral tests were analyzed with Kruskal-Wallis, Dunn's test, except for CFC. CFC was analyzed with one-way ANOVA, Tukey post-hoc.

Behavioral Tests	Parameters	3-MPI	6-MPI	9-MPI
OFA	Distance covered	-	H (3, 40) = 0.1629, $p = 0.922$	H (3, 33) = 2.033, $p = 0.362$
	Speed (cm/s)	-	H (3, 40) = 0.1402, $p = 0.932$	H (3, 33) = 1.914, $p = 0.384$
	Time in center (s)	-	H (3, 40) = 7.115, $p = 0.029$ *	H (3, 33) = 2.833, $p = 0.243$
EPM	Time in open arm (s)	-	H (3, 40) = 2.449, $p = 0.294$	-
	Time in closed arm (s)	-	H (3, 40) = 1.380, $p = 0.502$	-
	Ratio of open vs. closed	-	H (3, 40) = 2.794, $p = 0.247$	-
CFC [1]	Contextual	-	F (2, 21) = 1.967, $p = 0.165$	F (2, 30) = 1.860, $p = 0.173$
	Cued	-	F (2, 21) = 0.195, $p = 0.824$	F (2, 30) = 5.497, $p = 0.009$ **
Pole Test	Time to descend pole (s)	-	H (3, 40) = 10.68, $p = 0.005$ **	H (3, 33) = 3.061, $p = 0.216$
	Time to T-turn (s)	-	H (3, 40) = 7.119, $p = 0.029$ *	H (3, 33) = 4.268, $p = 0.118$
Beam Walk	Time to cross beam (s)	-	H (3, 40) = 0.385, $p = 0.825$	H (3, 33) = 2.466, $p = 0.291$
	Number of slips (s)	-	H (3, 40) = 1.230, $p = 0.541$	H (3, 33) = 0.088, $p = 0.957$

[1] Analyzed with one-way ANOVA, Tukey post-hoc

[*] Indicates statistical significance ($p < 0.05$)

[**] Indicates statistical significance ($p < 0.01$)

Supplementary Table S2. Behavioral statistics of 3-, 6-, and 9-MPI hThy1-αsyn overexpressing human tau. Statistics for each behavioral test and their corresponding measured parameters (open field analysis (OFA), elevated plus maze (EPM), contextual/cued fear conditioning (CFC)). All behavioral tests were analyzed with Kruskal-Wallis, Dunn's test, except for CFC. CFC was analyzed with one-way ANOVA, Tukey post-hoc.

Behavioral Tests	Parameters	3-MPI	6-MPI	9-MPI
OFA	Distance covered	H (3, 36) = 2.755, $p = 0.252$	H (3, 26) = 0.712, $p = 0.701$	H (3, 28) = 5.044, $p = 0.080$
	Speed (cm/s)	H (3, 37) = 2.864, $p = 0.239$	H (3, 26) = 0.673, $p = 0.714$	H (3, 28) = 5.233, $p = 0.073$
	Time in center (s)	H (3, 36) = 2.748, $p = 0.253$	H (3, 26) = 1.001, $p = 0.606$	H (3, 28) = 1.787, $p = 0.410$
EPM	Time in open arm (s)	H (3, 36) = 3.030, $p = 0.212$	H (3, 26) = 2.293, $p = 0.318$	H (3, 28) = 4.879, $p = 0.087$
	Time in closed arm (s)	H (3, 36) = 9.476, $p = 0.009$ **	H (3, 26) = 1.726, $p = 0.422$	H (3, 28) = 2.723, $p = 0.256$
	Ratio of open vs. closed	H (3, 36) = 3.030, $p = 0.220$	H (3, 26) = 2.293, $p = 0.318$	H (3, 28) = 4.879, $p = 0.087$
CFC [1]	Contextual	F (2, 33) = 1.718, $p = 0.195$	F (2, 23) = 1.298, $p = 0.292$	F (2, 25) = 1.177, $p = 0.325$
	Cued	F (2, 33) = 1.772, $p = 0.186$	F (2, 23) = 1.394, $p = 0.268$	F (2, 25) = 0.087, $p = 0.917$
Pole Test	Time to descend pole (s)	H (3, 36) = 8.415, $p = 0.015$ *	H (3, 26) = 1.798, $p = 0.407$	H (3, 27) = 2.140, $p = 0.343$
	Time to T-turn (s)	H (3, 36) = 8.431, $p = 0.015$ *	H (3, 26) = 2.487, $p = 0.288$	H (3, 27) = 1.998, $p = 0.368$
Beam Walk	Time to cross beam (s)	H (3, 36) = 1.947, $p = 0.378$	H (3, 26) = 3.389, $p = 0.184$	H (3, 27) = 4.378, $p = 0.112$
	Number of slips (s)	H (3, 36) = 5.366, $p = 0.068$	H (3, 26) = 3.529, $p = 0.171$	H (3, 27) = 3.493, $p = 0.174$

[1] Analyzed with one-way ANOVA, Tukey post-hoc

* Indicates statistical significance ($p < 0.05$)

** Indicates statistical significance ($p < 0.01$)

Supplementary Table S3. Behavioral statistics of 3-MPI of APP/PS1 overexpressing human αsyn. Statistics for each behavioral test and their corresponding measured parameters (open field analysis (OFA), elevated plus maze (EPM), contextual/cued fear conditioning (CFC)). All behavioral tests were analyzed with Kruskal-Wallis, Dunn's test, except for CFC. CFC was analyzed with one-way ANOVA, Tukey post-hoc.

Behavioral Tests	Parameters	3-MPI	6-MPI	9-MPI
OFA	Distance covered	H (3, 20) = 4.960, $p = 0.080$	-	-
	Speed (cm/s)	H (3, 20) = 5.069, $p = 0.074$	-	-
	Time in center (s)	H (3, 20) = 4.052, $p = 0.132$	-	-
EPM	Time in open arm (s)	H (3, 20) = 0.863, $p = 0.668$	-	-
	Time in closed arm (s)	H (3, 20) = 1.930, $p = 0.398$	-	-
	Ratio of open vs. closed	H (3, 20) = 1.000, $p = 0.627$	-	-
CFC [1]	Contextual	F (2, 17) = 0.234, $p = 0.794$	-	-
	Cued	F (2, 17) = 0.240, $p = 0.789$	-	-
Pole Test	Time to descend pole (s)	H (3, 20) = 1.856, $p = 0.414$	-	-
	Time to T-turn (s)	H (3, 20) = 2.066, $p = 0.372$	-	-
Beam Walk	Time to cross beam (s)	H (3, 20) = 2.065, $p = 0.373$	-	-
	Number of slips (s)	H (3, 20) = 0.549, $p = 0.782$	-	-

[1] Analyzed with one-way ANOVA, Tukey post-hoc

[*] Indicates statistical significance ($p < 0.05$)

[**] Indicates statistical significance ($p < 0.01$)

Supplementary Figure S1

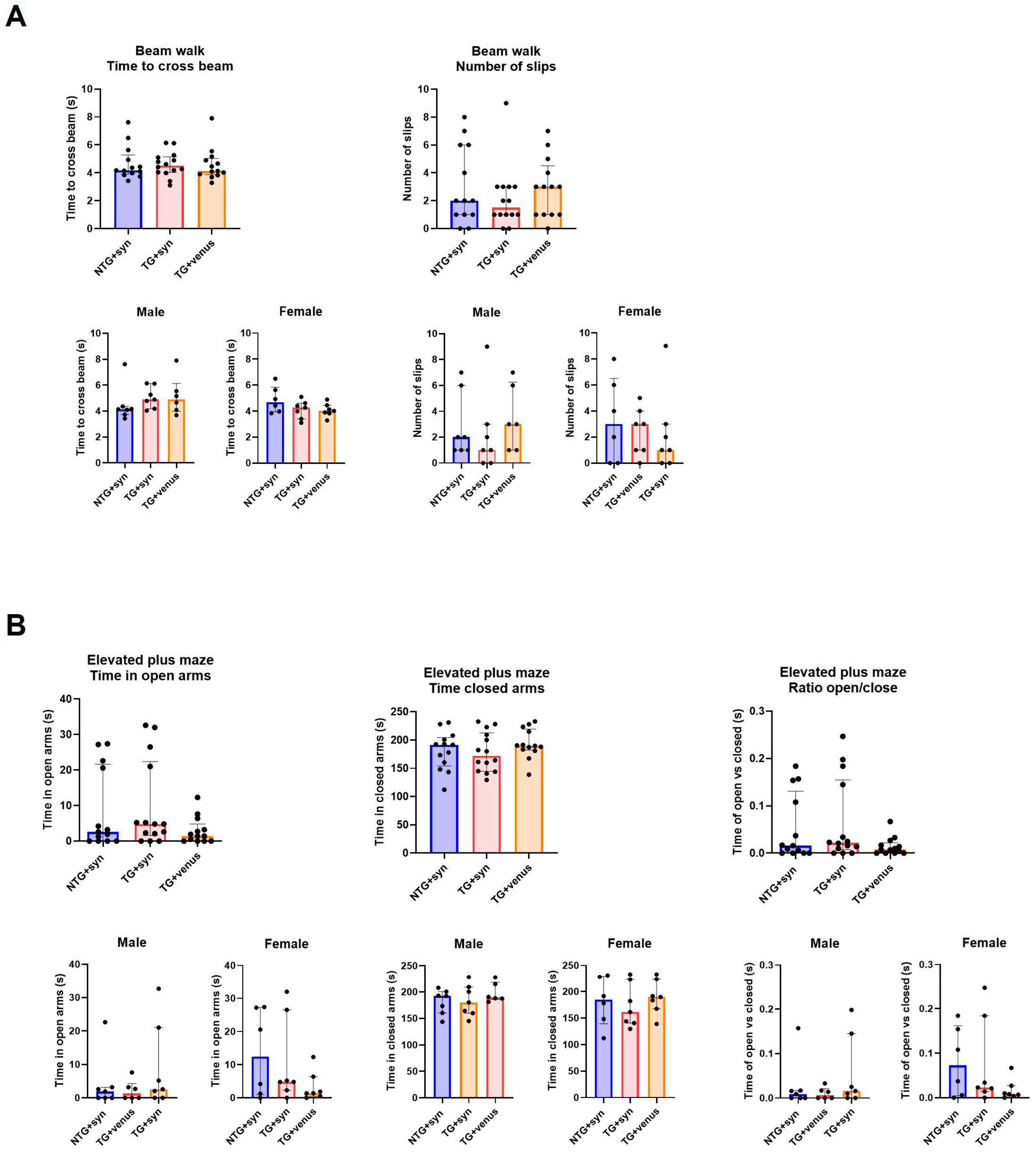

Supplementary Figure S1. Mobility and anxiety-like assessment of hTau mice at 6-MPI overexpressing human αsyn. (**A**) Motor behavior assessed in the time to cross beam (left) and average number of slips (right). No significant differences were seen in all groups [n= ± 30 per MPI group, Kruskal-Wallis, Dunn's test]. (**B**) No significant differences were observed in all parameters for the elevated plus maze for time in open arms (left), time in closed arms (middle), and time in open vs. closed (right) [n= ± 30, Kruskal-Wallis, Dunn's test].

Supplementary Figure S2

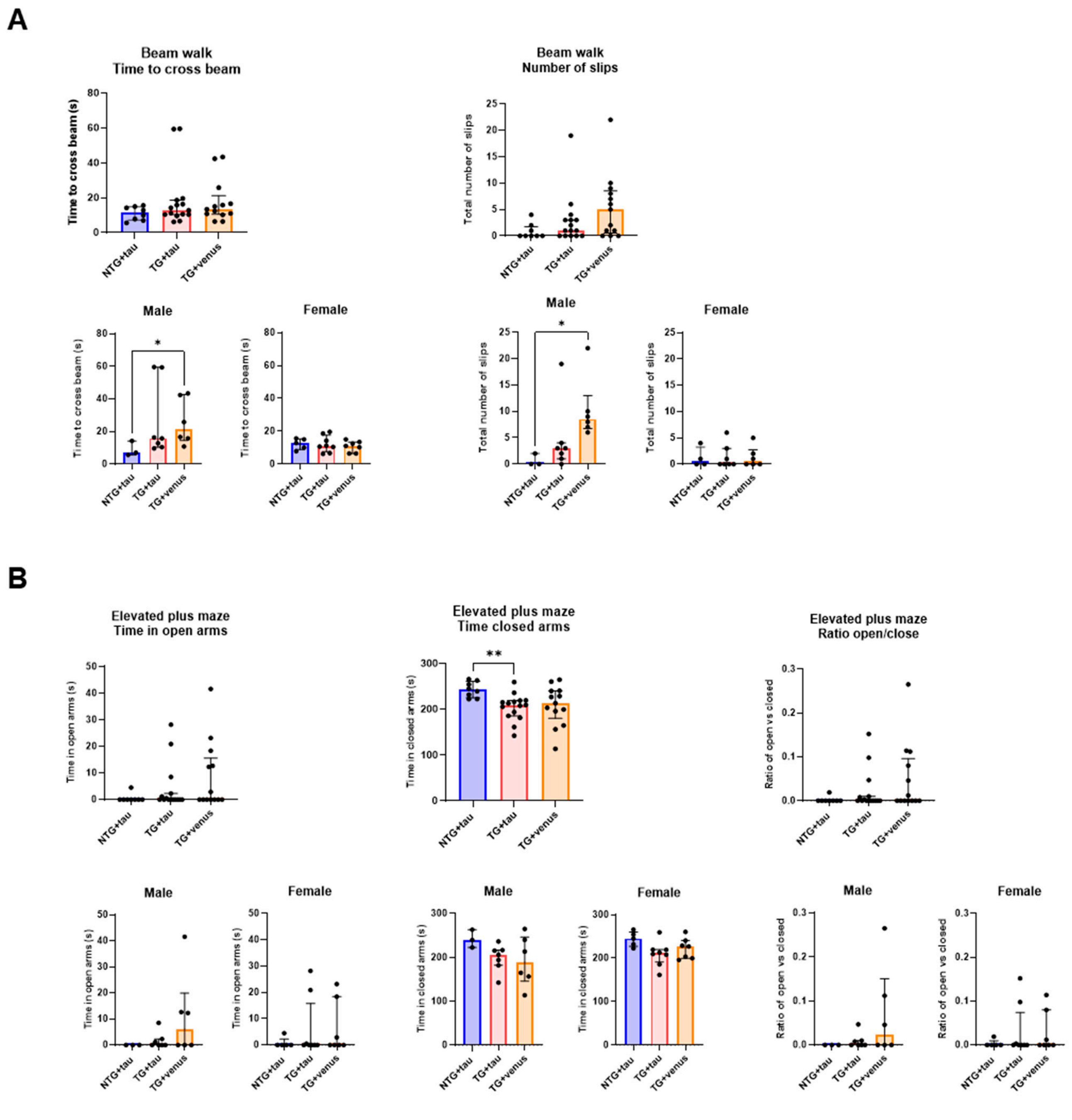

Supplementary Figure S2. Motor behavior and anxiety-like evaluation of hThy1- αsyn mice at 3-MPI overexpressing human tau (**A**) Motor behavior assessed in the beam walk test showing the time to cross the beam (left) and number of slips (right). Overall, no significant differences were observed, however, NTG+tau males demonstrate faster cross time and a significantly lower number of slips compared to their male TG+venus counterparts [n= ± 30, Kruskal-Wallis, Dunn's test, *$p < 0.05$]. (**B**) Anxiety-like behavior was assessed in the EPM, with no statistical differences in, time in open arms, time in closed arms, and the ratio of time spent in open vs. closed arms (left, middle, right) [n= ± 30, Kruskal-Wallis, **$p < 0.01$].

Supplementary Figure S3

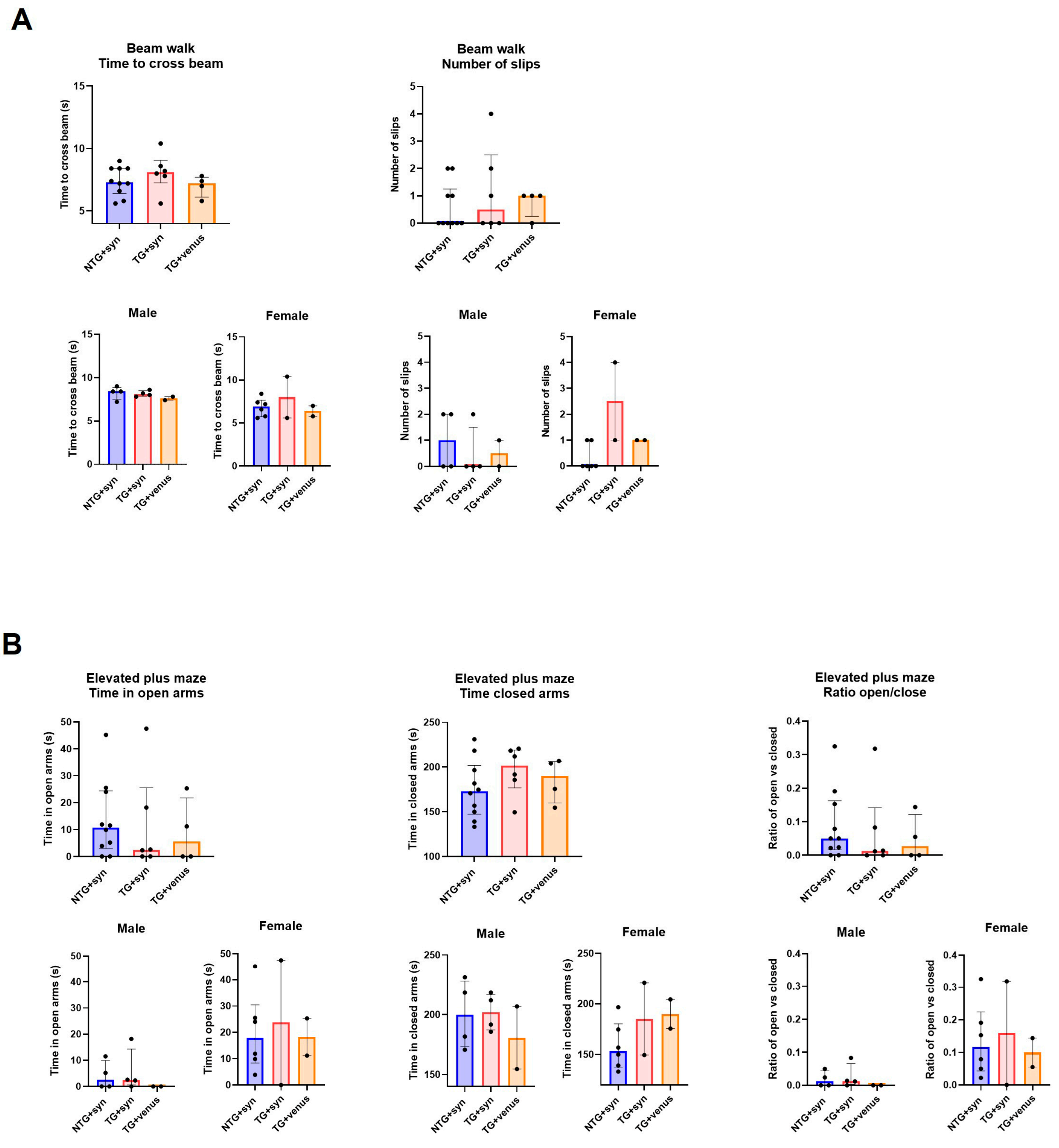

Supplementary Figure S3. Assessment of 3-MPI APP/PS1 overexpressing human αsyn on anxiety and mobility (**A**) No significant differences were observed in time to cross beam (left) and number of slips (right) [n= ± 30, Kruskal-Wallis, Dunn's test]. (**B**) No significant differences were observed in all parameters for the elevated plus maze for time in open arms (left), time in closed arms (middle), and time in open vs. closed (right) [n= ± 30, Kruskal-Wallis, Dunn's test,].
